# Association between Nutrition Policies and Student Body Mass Index

**DOI:** 10.3390/nu13010013

**Published:** 2020-12-23

**Authors:** Marlene B. Schwartz, Julien Leider, Juliana F. W. Cohen, Lindsey Turner, Jamie F. Chriqui

**Affiliations:** 1 Rudd Center for Food Policy and Obesity, Department of Human Development and Family Sciences, University of Connecticut, 1 Constitution Plaza, Hartford, CT 06103, USA; 2 Institute for Health Research and Policy, University of Illinois Chicago, Chicago, IL 60608, USA; jleide2@uic.edu (J.L.); jchriqui@uic.edu (J.F.C.); 3 Department of Public Health and Nutrition, Merrimack College, 315 Turnpike Street, North Andover, MA 01845, USA; cohenj@merrimack.edu; 4 Department of Nutrition, Harvard T.H. Chan School of Public Health, 677 Huntington Ave, Boston, MA 02115, USA; 5 College of Education, Boise State University, 1910 University Drive, Boise, ID 83725, USA; lindseyturner1@boisestate.edu; 6 Division of Health Policy and Administration, School of Public Health, University of Illinois Chicago, 1603 W. Taylor St, Chicago, IL 60612, USA

**Keywords:** school, nutrition, competitive foods, childhood obesity, school nutrition policies, state law

## Abstract

In response to concerns about childhood obesity, many US states have implemented policies to limit the sale of unhealthy foods and beverages (e.g., snacks, desserts, and sugary drinks) sold in competition with school meal programs (i.e., competitive foods) in order to improve the nutritional environment of schools and support student health. This study measured state-level competitive food and beverage policies that require foods and beverages sold in à la carte lines, vending machines, and school stores to meet strong nutrition standards and tested the hypothesis that students living in states with stronger laws would have lower body mass index (BMI)-for-age percentiles. BMI data from a national sample of 1625 students attending 284 schools from the School Nutrition and Meal Cost Study were linked to state laws coded as part of the National Wellness Policy Study. A survey-adjusted linear regression model accounting for student and school-level characteristics showed that stronger state nutrition policies were associated with lower student BMI scores (coefficient: −0.06, 95% CI: −0.12, −0.00). Additional models indicated that stronger state policies were significantly associated with fewer unhealthy foods and beverages available in schools. These findings suggest that strong regulations on competitive foods and beverages may lead to improvements in the nutritional quality of the school environment and student BMI. Thus, current federal standards regulating snacks in US schools (i.e., Smart Snacks) are an important element of a comprehensive strategy to improve the school nutrition environment and reduce rates of childhood obesity.

## 1. Introduction

In the United States, most schools sell “competitive” foods and beverages, which are described in this way because they are sold outside of the school meal program [1]. These products are typically sold as à la carte options in cafeterias, in vending machines, and in school stores. Historically, competitive foods have been calorically dense options that are low in nutrients and high in saturated fat, sodium, and sugar, such as snacks (e.g., potato chips), desserts (e.g., cookies, ice cream), and sugary beverages (e.g., sports drinks) [2,3].

The availability of unhealthy competitive foods and beverages in schools has been shown to be detrimental to students’ diet quality [4,5,6] and, in some studies, to increase the risk of obesity [6,7,8]. For example, Dighe and colleagues examined the relationship between the school nutrition and physical activity environment and student BMI in a sample of students from low-income areas of New Jersey [8]. They found a significant association between the number of unhealthy items sold in school vending machines and student BMI z-scores [8].

In response to concerns about the harm associated with competitive foods and beverages, there have been considerable efforts to limit their availability in schools through district-, state-, and federal-level policies. Beginning in 2006, federal regulations required all local education agencies (typically school districts) to create written policies that addressed, among other things, nutrition standards for competitive foods [9]. Because these policies were written locally, they were quite variable and only 45% of districts adopted strong (i.e., definitively required) competitive food and beverage policies the first year (2006–2007) [10]. Concurrently, states began enacting laws to regulate competitive food and beverages in schools. Between school years 2006–2007 and 2013–2014, the proportion of states with required nutrition standards for competitive foods and beverages increased from 24.84% to 41.18% [11]. At the federal level, the 2010 Healthy, Hunger-Free Kids Act (HHFKA) required the USDA to develop new competitive food and beverage standards. In response, the USDA developed and released the “Smart Snacks” criteria in 2013 [12]. With the release of Smart Snacks, the USDA significantly strengthened the federal nutrition standards for competitive foods and beverages and brought them into alignment with current evidence-based recommendations. The 2014–2015 school year was the first year that Smart Snacks were implemented nationally.

There is accumulating evidence that implementing strong competitive food and beverage nutrition policies positively influences the school nutrition environment and is associated with healthier student diets [13]. For example, a state law in Massachusetts led to decreases in the availability of unhealthy competitive foods and beverages [14,15] and significant improvements in student diets [16]. One multi-state study found that students living in states with strong competitive food and beverage policies consumed fewer calories from solid fats and added sugars than students living in states without these laws [17]. A second multi-state study found that secondary students living in the state with the strongest competitive food law in the country consumed fewer calories per week than students in 14 states with no competitive food laws [18].

The above studies found a relationship between school nutrition policies and student diet quality; however, to date, only a few studies have found a relationship between school nutrition policies and student BMI. One cross-sectional study of students from military families found an association between the children’s BMI z-scores and the strength of the competitive food and beverage policies in the states where their families lived [19]. A longitudinal study found that students in states with stronger competitive food laws gained less weight between fifth and eighth grade than students in states without strong laws [20]. More recently, Kenney and colleagues used pooled cross-sectional data from the National Survey of Children’s Health to assess childhood obesity trends before and after the implementation of the HHFKA [21]. Although there was not a shift in childhood obesity rates overall after the HHFKA was implemented, the risk of obesity significantly declined among low-income students for each year following the implementation of the HHFKA.

To further explore how competitive food and beverage policies are associated with student weight outcomes, the current study examines state-level competitive food and beverage policies and student BMI-for-age percentiles. The primary objective was to test the hypothesis that strong state-level policies will be associated with lower student BMI-for-age percentiles. Secondary objectives were to evaluate (1) the relationship between state laws and the school nutrition environment (i.e., the number of unhealthy foods and beverages available); (2) the relationship between the school nutrition environment and student BMI-for-age percentiles; and (3) the relationship of state laws and the school environment with student BMI-for-age percentiles.

## 2. Materials and Methods

### 2.1. Data and Design

School Nutrition and Meal Cost Study (SNMCS) data were used to assess student BMI and the availability of competitive foods and beverages. Using a two-stage sampling approach, schools were sampled within school food authorities (the entities responsible for administering school meal programs locally) and students were randomly sampled from students enrolled at the sampled schools. The student-level SNMCS data are nationally representative of students enrolled in public, non-charter schools that participated in the National School Lunch Program. Data collection occurred in January–June 2015 (2014–2015 school year). The methodology report for the SNMCS provides a detailed description of the design and the procedures used for sampling, recruitment, data collection, and data processing [22]. Additional details about the collection and analysis of the data used in this paper are available in Volumes 1 and 4 of the SNMCS final report [23,24]. Mathematica Policy Research provided de-identified SNMCS data to the University of Illinois Chicago for analyses. The SNMCS data were linked with state law data from the National Wellness Policy Study [11]. This study was deemed to “not involve human subjects” by the University of Illinois Chicago Institutional Review Board (protocol #2020-0448).

### 2.2. Measures

#### 2.2.1. Student BMI

Student BMI-for-age percentiles were computed as part of SNMCS based on age, sex, and measured height and weight according to Centers for Disease Control and Prevention guidelines. The BMI measure was set to missing for implausible values of height, weight, and age.

#### 2.2.2. School Competitive Food and Beverage Environments

Data on available competitive foods and beverages were obtained from the SNMCS Vending Machine, Other Sources of Food/Beverages, and À la Carte Checklists. The Vending Machine Checklist asked about a number of specific items in vending machines available to students, including before or after school, with a separate checklist for each machine. For schools that had vending machines available to students, respondents were asked to indicate the number of slots or buttons allocated to each item. These responses were used to compute indicators for whether each item was available in any machine, treating items within a machine as not available where the given item was left blank but information was filled in for other items. The Other Sources of Food/Beverages Checklist asked respondents to indicate whether a list of items were available to students (including before or after school) in four different venues, including school stores, snack bars/food carts/kiosks, fundraisers, and other venues. For each item, an indicator was computed for whether the item was available in school stores. Finally, the À la Carte Checklist asked respondents to indicate whether a list of items were available to students at breakfast or lunch. Because not all schools in the analytical sample offered breakfast, indicators were computed for whether each item was available at lunch specifically; indicators were set to missing for non-milk items in cases where the respondent indicated à la carte items other than milk were served but no specific non-milk items were selected.

Based on these computed indicators, the total number of unhealthy foods and beverages in vending machines, school stores, and à la carte was computed. For this purpose, all items classified as baked goods/desserts, frozen/dairy desserts, or snacks on the À la Carte Checklist were counted as unhealthy, as were deep-fried French fries (across all three venues). For beverages, the Smart Snacks criteria were applied, so the following items were counted as unhealthy: whole or reduced-fat white milk; whole, reduced-fat, or low-fat flavored milk; diet carbonated soft drinks (to be consistent with Smart Snacks, these only counted as unhealthy at the elementary and middle school levels); regular carbonated soft drinks; juice drinks and other sweetened drinks; sports drinks; energy drinks; hot or cold chocolate drinks; and hot or cold coffee or tea [12]. A total of 102 potential unhealthy items were assessed across venues.

#### 2.2.3. Policy Measures

The methods used for collecting and coding state laws for all 50 states and the District of Columbia as part of the National Wellness Policy Study have been described in detail previously [11]. Briefly, laws were compiled and coded by an attorney who served as the lead legal researcher and a second coder, both of whom have over a decade of experience in compiling and coding these laws. All laws were double-coded and a consensus coding was reached. Laws that are required are considered “strong”, whereas laws that are not required are encouraged and therefore are considered “weak” because they are not enforceable.

The analyses in this paper utilized a measure of the extent to which state laws included specific requirements for competitive foods and beverages available in vending machines, school stores, and à la carte. Specifically, variables NS1–11 (overarching competitive food variables) and venue-specific variables NS12–34 (that addressed specific nutrient standards for foods and beverages sold in each location of sale) were categorized as either including specific requirements (level 2 coding or greater) or including only weak/suggested/encouraged language (level 1 coding), or not being addressed (coded as 0). The percentage of variables with specific requirements was computed to generate a score ranging from 0 to 100, summarizing the strength of state laws in this area.

#### 2.2.4. Control Measures

School and student-level characteristics were obtained from the SNMCS and public use datasets from the National Center for Education Statistics [25]. School-level characteristics included the racial/ethnic distribution of each school’s students (≥50% non-Hispanic White, ≥50% non-Hispanic Black, ≥50% Hispanic, and mixed), the percentage of students eligible for free/reduced-price lunch (FRPL) (≤37.42%, >37.42–63.37%, and >63.37%, categorized by tertile), urbanicity (urban, suburban, and rural), size (<500, 500–999, and ≥1000 students), and Census region (West, Midwest, South, and Northeast) [26]. Student-level characteristics included grade level, sex, race/ethnicity (non-Hispanic white, non-Hispanic Black, Hispanic, and other, including multi-racial), and household income as a percentage of the poverty level (≤130%, >130–185%, and >185%, categorized based on thresholds used for determining FRPL eligibility).

### 2.3. Study Sample

**Figure 1:**
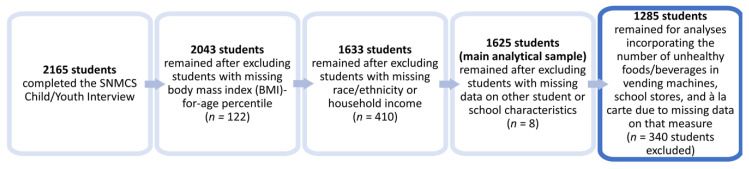
Sample size changes due to missing data.

The SNMCS Child/Youth Interview (63.6% weighted response rate) was completed by 2165 students [22]; however, the sample size decreased due to missing data (see Figure 1). Specifically, 122 students were missing data on their BMI-for-age percentile, 410 were missing data on their race/ethnicity or household income, and 8 were missing data on other school or student-level characteristics, leaving an analytical sample of 1625 students in 284 schools in 30 states and the District of Columbia (DC). The analytical sample was statistically different from the full respondent sample in terms of school size (fewer large schools in the analytical sample), student grade (fewer students in grades 7–12 in the analytical sample), and household income (fewer students with household income ≤130% of the poverty level in the analytical sample). Due to missing data on the number of unhealthy foods and beverages in vending machines, school stores, and à la carte, analyses which incorporated this measure were limited to 1285 students in 226 schools in 30 states and DC. This sample was statistically different from the full respondent sample in terms of student grade (fewer students in grades 7–12 in the analytical sample) and household income (more students with household income >185% of poverty in the analytical sample). Because of differences in the sample employed, statistics in this paper may differ from those in the SNMCS report [24].

### 2.4. Data Analyses

A multivariable linear regression model was computed examining the association of state law and school and student characteristics with student BMI-for-age percentiles. To further explore the extent to which state law might be associated with student BMI-for-age percentiles through the number of unhealthy foods and beverages available in vending machines, school stores, and à la carte, separate analyses were conducted linking: (1) state law to the number of unhealthy foods and beverages, controlling for school characteristics, (2) the number of unhealthy foods and beverages to student BMI-for-age percentiles, controlling for school and student characteristics, and (3) both state law and the number of unhealthy foods and beverages to student BMI-for-age percentiles, controlling for school and student characteristics. Due to the high prevalence (19.3%) of zero values for the number of unhealthy foods and beverages, analysis (1) was computed as a multivariable zero-inflated Poisson model, with a logistic regression model for excess zeroes and a Poisson model for the number of unhealthy foods and beverages. Analyses (2) and (3) were computed as multivariable linear regression models. As noted, there was some variation in the measures included in analyses (1), (2), and (3); to ensure consistency in the sample employed, these three analyses were conducted for the sample for which all relevant measures were non-missing.

Analyses were conducted in Stata/SE (version 15.1, StataCorp LP, College Station, TX, USA; 2016) accounting for the survey design and weights, treating strata with a single sampling unit as certainty units.

## 3. Results

### 3.1. Sample Characteristics

The analytical sample of students was socioeconomically and racially/ethnically diverse and included students of all grade levels, from schools of diverse geographies, sizes, and socioeconomic and racial/ethnic distributions (Table 1). The mean BMI-for-age percentile was 66.1, although the interquartile range extended from 45.3 to 91.0 (not shown in tables). Students were in states that had strong laws on 33.2% of policies evaluated for competitive foods and beverages in vending machines, school stores, and à la carte, on average, and were enrolled in schools with 7.5 unhealthy foods and beverages available in vending machines, school stores, and à la carte, on average.

### 3.2. Relationship between State Laws and Student BMI-for-Age Percentiles

Table 2 shows the results of a multivariable linear regression model examining the association of state law and school and student characteristics with student BMI-for-age percentiles. Having stronger state laws on competitive foods and beverages in vending machines, school stores, and à la carte was associated with lower student BMI-for-age percentiles (coeff.: −0.06, 95% CI: −0.12, −0.00), corresponding to an adjusted mean BMI-for-age percentile of 68.0 with a state law strength score of 0 and an adjusted mean of 62.1 with a state law strength score of 100 where required policies are in place for all items considered. The results also show that non-Hispanic Black (coeff: 10.22, 95% CI: 4.54, 15.90) and Hispanic (coeff.: 6.44, 95% CI: 1.21, 11.68) students had higher BMI-for-age percentiles compared to non-Hispanic White students, as did students in schools with medium (coeff.: 6.90, 95% CI: 1.37, 12.42) and high (coeff.: 15.75, 95% CI: 10.21, 21.28) FRPL eligibility rates compared to those with low FRPL eligibility rates, and students in suburban compared to urban schools (coeff.: 5.16, 95% CI: 0.19, 10.12). Students in schools with a mixed racial/ethnic distribution had lower BMI-for-age percentiles than those in majority non-Hispanic White schools (coeff.: −5.90, 95% CI: −11.21, −0.59).

### 3.3. Relationship between State Laws and Availability of Unhealthy Competitive Foods and Beverages in Schools

Table 3 shows the results of multivariable regressions examining the extent to which the association between state law and student BMI-for-age percentiles occurred through changes in the number of unhealthy competitive foods and beverages available in schools. Based on a zero-inflated Poisson model, stronger state laws were associated with higher odds of not having any unhealthy competitive foods and beverages available (OR: 1.02, 95% CI: 1.00, 1.04), and a lower number of unhealthy competitive foods and beverages available (IRR: 0.99, 95% CI: 0.99, 1.00). Schools with a medium as opposed to low rate of FRPL eligibility were more likely not to have any unhealthy competitive foods and beverages (OR: 4.62, 95% CI: 1.24, 17.24), and large schools were less likely than small schools not to have any unhealthy competitive foods and beverages (OR: 0.01, 95% CI: 0.00, 0.15) and had more unhealthy competitive foods and beverages (IRR: 2.25, 95% CI: 1.56, 3.24). Schools in the South were less likely to have no unhealthy competitive foods and beverages than those in the West (OR: 0.25, 95% CI: 0.07, 0.92), while schools in the Northeast had more unhealthy competitive foods and beverages than those in the West (IRR: 1.68, 95% CI: 1.06, 2.67).

### 3.4. Relationship between the Availability of Unhealthy Competitive Foods and Beverages, State Law, and Student BMI-for-Age Percentiles

Table 3 also presents two additional multivariable linear regression models. In the first one, the number of unhealthy competitive foods and beverages available was not associated with student BMI-for-age percentiles, although it approached significance (coeff.: 0.27, 95% CI: −0.04, 0.59) and the coefficient was positive, indicating that a larger number of unhealthy competitive foods and beverages would be associated with higher student BMI-for-age percentiles. Neither the number of unhealthy competitive foods and beverages nor the state law strength score were significantly associated with student BMI-for-age percentiles when both were included in the model. Associations between other school and student characteristics and student BMI-for-age percentiles from these two models were similar to those in the primary specification in Table 2.

## 4. Discussions

This study found that stronger state competitive food and beverage laws were associated with significantly lower student BMI-for-age percentiles in a nationally representative sample of students from 1st through 12th grade. To further understand how state laws may influence BMI, we examined the relationship between strength of the laws and the school food environment and found that, consistent with our hypothesis, schools in states with stronger laws had fewer unhealthy competitive foods and beverages available for sale. These findings suggest that stronger laws lead to healthier environments, which may represent one path through which these laws are associated with lower BMI.

In our subsequent models, we measured the direct relationship between the availability of unhealthy competitive foods and beverages and student BMI-for-age percentiles. Although the relationship was in the expected direction, it did not reach statistical significance (*p* < 0.10). Finally, we tested a model that included both state laws and the number of unhealthy competitive foods and beverages as predictors of BMI-for-age percentiles and, again, the results were in the expected direction, but not statistically significant. Due to missing data, our sample for all analyses incorporating the number of available unhealthy competitive foods and beverages was only 1285 compared to 1625 for analyses not incorporating that measure. Future research tracking a larger sample over time may be needed to establish the path through which strong laws influence student BMI.

It is notable that these data were collected in 2014–2015, which was the first year of Smart Snacks implementation. The fact that there was still quite a large average number of unhealthy competitive foods and beverages available suggests that some schools had not yet fully adopted these new nutrition standards. Our finding that schools in states with stronger competitive food laws had fewer unhealthy competitive foods is consistent with the idea that when new federal policies are introduced, schools located in states with preexisting policies consistent with the new standards may have an advantage in reaching compliance due to established frameworks and guidance. Indeed, in a study mentioned earlier, Turner and colleagues identified which states did or did not have state-level competitive food laws that met the new federal Smart Snack standards. Then, they used student-level SNMCS dietary data from 2014–2015 to compare the dietary intake of students living in states with and without these laws. They found that students in states with laws that were consistent with Smart Snacks reported eating significantly lower amounts of added sugars and solid fats than students living in other states [17].

Although the current study was conducted in the US, the findings may be relevant to other countries that are engaged in policy efforts to regulate canteens and other sources of unhealthy foods and beverages in schools [27]. For example, Australian states and territories have school nutrition policies that regulate school canteens based on a “traffic light” classification system [28]. However, recent evidence from secondary schools in Australia suggests that it is easier to comply with the requirement to provide a majority of “green” items than the recommendation to eliminate the “red” items [29]. There have also been considerable efforts in England to improve school food, including the current School Food Standards that apply to school meals and food available outside of meals [30,31]. Although progress has been made, a 2017 evaluation by the Jamie Oliver Food Foundation found a lack of monitoring and enforcing of these standards in secondary schools [32]. Clearly, many countries face the challenge of limiting student access to unhealthy foods at school and the finding that strong policies are associated with lower BMI levels may be useful in advocating for targeted efforts to implement nutrition policies outside of the US.

The findings from the present study are consistent with a recent Canadian study on student BMI and exposure to strong school food policies [33]. In Canada, there is not a national school lunch program; instead, provincial/territorial governments set their own school nutrition policies. Between 2005 and 2011, six provinces chose to ban “junk food” in schools, while the remainder did not. In 2017, Leonard evaluated this natural experiment and analyzed student BMI data from the Canadian Community Health Survey while considering the number of years that the student had been in a province with or without a ban. The findings indicated that exposure to five or more years of a “junk food ban” was associated with a significantly lower BMI [33].

The results of the present study suggest that state laws may play an integral role in enhancing compliance with federal school nutrition regulations in the US, which improves the school nutrition environment and can, in turn, have important health implications for children. Reducing the risk of obesity remains crucially important to protect children’s short- and long-term health. In addition to the association of childhood obesity with poor health outcomes during childhood, it also increases the risk of obesity and chronic diseases including cardiovascular disease, diabetes, and cancer as an adult [34,35].

### Study Limitations

This study was subject to several limitations. First, detailed nutritional data on competitive foods and beverages available in schools were not available and therefore the analyses were limited to broad item descriptions from the SNMCS À la Carte, Vending Machine, and Other Sources of Food/Beverages Checklists. Second, there was also a fairly large amount of missing data on these measures. As a result, this study’s ability to assess the extent to which state laws were associated with BMI through this pathway were limited by a smaller sample and less statistical power. Third, because the data were cross-sectional, causality cannot be established. Finally, there were differences on some characteristics (including household income, student grade, and school size) between the full sample and the analytical samples after excluding cases with missing data, which may limit the generalizability of this study’s findings. Strengths of this study included the use of student BMI-for-age percentiles based on objectively measured height and weight, the use of objectively coded data on relevant state laws, and the use of data from a nationally representative sample of students in 30 states and the District of Columbia. Future studies should examine the detailed availability of competitive foods and beverages in schools and the impact on consumption and BMI over time.

## 5. Conclusions

This study suggests that the combined impact of strong federal and state competitive food and beverage standards is associated with lower student BMI and that this may be in part due to the association between the stronger laws and the reduced availability of unhealthy foods and beverages available in schools. This study provides evidence that lawmakers should prioritize protecting—and not weakening—the Smart Snack standards. Further, states that do not currently have competitive food laws that reflect or go beyond the federal regulations should strongly consider enacting them in order to ensure that the children in their state have the benefit of attending schools where they are protected from exposure to unhealthy foods and beverages.

## Figures and Tables

**Table 1:** Analytic sample characteristics.

Variable	% or Mean (95% CI)
BMI-for-age percentile (Mean)	66.1 (63.9, 68.2)
State law strength score for competitive foods and beverages in vending machines, school stores, and à la carte (0–100) (Mean)	33.2 (25.6, 40.8)
Number of unhealthy foods and beverages in vending machines, school stores, and à la carte (Mean)	7.5 (5.9, 9.1)
Race/Ethnicity of Students in School	
≥50% Non-Hispanic White	60.6 (50.3, 69.9)
≥50% Non-Hispanic Black	6.8 (3.0, 14.7)
≥50% Hispanic	15.2 (9.8, 22.9)
Mixed	17.4 (12.1, 24.5)
Free/Reduced-Price Lunch Eligibility Rate Tertiles	
Low (0.00–37.42)	35.3 (26.3, 45.5)
Medium (>37.42–63.37)	30.2 (22.6, 39.2)
High (>63.37–100.00)	34.5 (26.0, 44.0)
School Urbanicity	
Urban	24.1 (16.8, 33.2)
Suburban	49.1 (38.6, 59.7)
Rural	26.8 (17.8, 38.3)
School Size	
Small (fewer than 500 students)	25.3 (18.6, 33.4)
Medium (500 to 999 students)	45.9 (37.5, 54.5)
Large (1000 or more students)	28.9 (22.5, 36.2)
Census Region	
West	18.2 (11.0, 28.8)
Midwest	25.1 (16.1, 36.9)
South	44.0 (32.7, 55.9)
Northeast	12.7 (6.6, 23.1)
Student grade	
1	8.3 (6.4, 10.8)
2	9.9 (8.1, 11.9)
3	10.9 (8.8, 13.5)
4	9.1 (7.1, 11.6)
5	8.8 (7.2, 10.7)
6	7.7 (5.9, 10.0)
7	7.0 (5.4, 9.1)
8	7.0 (5.6, 8.6)
9	9.8 (7.7, 12.5)
10	7.9 (6.2, 10.2)
11	8.4 (6.5, 10.8)
12	5.3 (3.9, 7.1)
Student sex	
Male	49.3 (46.5, 52.1)
Female	50.7 (47.9, 53.5)
Student race/ethnicity	
White, non-Hispanic	51.3 (44.3, 58.3)
Black, non-Hispanic	13.5 (8.9, 19.9)
Hispanic	26.0 (20.7, 32.2)
Other (includes multi-racial)	9.2 (7.3, 11.4)
Household income as a percentage of poverty level	
≤130%	35.2 (30.1, 40.7)
>130–185%	9.9 (7.8, 12.5)
>185%	54.8 (48.8, 60.8)

Notes: Data are student-level and are survey-adjusted. *n* = 1625 students from the 2014–2015 School Nutrition and Meal Cost Study (SNMCS), except for the number of unhealthy foods and beverages in vending machines, school stores, and à la carte, for which *n* = 1285 due to missing data for that item. CI: confidence interval.

**Table 2:** Multivariable linear regression results for the association of state law and school and student characteristics with student BMI-for-age percentiles.

Variable	Coeff. (95% CI)
State law strength score for competitive foods and beverages in vending machines, school stores, and à la carte (0–100)	−0.06 * (−0.12, −0.00)
Race/Ethnicity of Students in School	
≥50% Non-Hispanic White	Referent
≥50% Non-Hispanic Black	−3.42 (−10.50, 3.66)
≥50% Hispanic	−3.44 (−8.98, 2.11)
Mixed	−5.90 * (−11.21, −0.59)
Free/Reduced-Price Lunch Eligibility Rate Tertiles	
Low (0.00–37.42)	Referent
Medium (>37.42–63.37)	6.90 * (1.37, 12.42)
High (>63.37–100.00)	15.75 *** (10.21, 21.28)
School Urbanicity	
Urban	Referent
Suburban	5.16 * (0.19, 10.12)
Rural	3.86 (−1.68, 9.40)
School Size	
Small (fewer than 500 students)	Referent
Medium (500 to 999 students)	0.08 (−4.01, 4.16)
Large (1000 or more students)	−0.01 (−5.39, 5.38)
Census Region	
West	Referent
Midwest	1.96 (−3.24, 7.17)
South	−0.28 (−5.31, 4.74)
Northeast	−0.49 (−5.35, 4.37)
Student grade (1–12)	0.31 (−0.25, 0.86)
Student sex	
Male	Referent
Female	2.30 (−1.24, 5.85)
Student race/ethnicity	
White, non-Hispanic	Referent
Black, non-Hispanic	10.22 *** (4.54, 15.90)
Hispanic	6.44 * (1.21, 11.68)
Other (includes multi-racial)	−3.83 (−9.92, 2.26)
Household income as a percentage of poverty level	
≤130%	−3.30 (−8.69, 2.10)
>130–185%	−5.20 (−12.20, 1.80)
>185%	Referent
Constant	54.29 *** (45.70, 62.87)
Adjusted mean student BMI-for-age percentile by state law strength score for competitive foods and beverages in vending machines, school stores, and à la carte	
State law strength score: 0	68.0
State law strength score: 100	62.1

Notes: *n* = 1625 students from the 2014–2015 School Nutrition and Meal Cost Study (SNMCS). Regressions were survey-adjusted and controlled for the variables shown. CI: confidence interval. * *p* < 0.05, *** *p* < 0.001.

**Table 3:** Multivariable regression results for the association of state law, the number of unhealthy competitive foods and beverages available, and school and student characteristics with student BMI-for-age percentiles.

Outcome:	Number of Unhealthy Competitive Foods and Beverages (Zero-Inflated Poisson Model)		BMI-for-Age Percentile	BMI-for-Age Percentile
Model type:	Logistic	Poisson	Linear	Linear
Variable	OR (95% CI)	IRR (95% CI)	Coeff. (95% CI)	Coeff. (95% CI)
State law strength score for competitive foods and beverages in vending machines, school stores, and à la carte (0–100)	1.02 * (1.00, 1.04)	0.99 *** (0.99, 1.00)	--	−0.04 (−0.11, 0.02)
Number of unhealthy foods and beverages in vending machines, school stores, and à la carte	--	--	0.27 + (−0.04, 0.59)	0.20 (−0.12, 0.51)
Race/Ethnicity of Students in School				
≥50% Non-Hispanic White	Referent	Referent	Referent	Referent
≥50% Non-Hispanic Black	5.07 (0.72, 35.84)	0.80 (0.38, 1.67)	0.36 (−7.53, 8.26)	−0.49 (−8.32, 7.35)
≥50% Hispanic	1.82 (0.36, 9.07)	0.68 + (0.44, 1.05)	−5.14 + (−10.55, 0.27)	−6.06 * (−11.77, −0.35)
Mixed	1.96 (0.46, 8.32)	1.16 (0.83, 1.62)	−6.57 * (−12.56, -0.58)	−6.89 * (-12.94, -0.84)
Free/Reduced-Price Lunch Eligibility Rate Tertiles				
Low (0.00–37.42)	Referent	Referent	Referent	Referent
Medium (>37.42–63.37)	4.62 * (1.24, 17.24)	1.03 (0.75, 1.40)	7.52 * (1.15, 13.89)	7.72 * (1.33, 14.12)
High (>63.37–100.00)	2.23 (0.62, 7.95)	0.80 (0.54, 1.20)	17.18 *** (11.22, 23.14)	17.24 *** (11.28, 23.20)
School Urbanicity				
Urban	Referent	Referent	Referent	Referent
Suburban	0.37 (0.10, 1.35)	1.17 (0.82, 1.66)	2.09 (−2.93, 7.12)	2.40 (−2.61, 7.41)
Rural	0.47 (0.09, 2.39)	1.14 (0.77, 1.70)	1.83 (−3.83, 7.49)	2.23 (−3.62, 8.07)
School Size				
Small (fewer than 500 students)	Referent	Referent	Referent	Referent
Medium (500 to 999 students)	0.91 (0.20, 4.16)	1.27 (0.89, 1.83)	1.21 (−3.34, 5.75)	1.55 (−2.97, 6.08)
Large (1000 or more students)	0.01 ** (0.00, 0.15)	2.25 *** (1.56, 3.24)	0.57 (−6.06, 7.21)	1.17 (−5.38, 7.72)
Census Region				
West	Referent	Referent	Referent	Referent
Midwest	0.25 (0.04, 1.64)	1.01 (0.64, 1.60)	3.08 (−2.59, 8.74)	2.25 (−3.46, 7.96)
South	0.25 * (0.07, 0.92)	1.24 (0.87, 1.78)	−0.06 (−5.63, 5.52)	−0.26 (−5.92, 5.41)
Northeast	0.93 (0.15, 5.58)	1.68 * (1.06, 2.67)	−2.89 (−8.78, 3.01)	−3.47 (−9.55, 2.61)
Student grade (1–12)	--	--	−0.05 (−0.77, 0.67)	−0.04 (−0.75, 0.67)
Student sex				
Male	--	--	Referent	Referent
Female	--	--	1.45 (−2.54, 5.45)	1.53 (−2.46, 5.52)
Student race/ethnicity				
White, non-Hispanic	--	--	Referent	Referent
Black, non-Hispanic	--	--	6.53 * (0.83, 12.24)	6.83 * (1.05, 12.60)
Hispanic	--	--	7.68 ** (2.06, 13.30)	7.65 ** (2.05, 13.25)
Other (includes multi-racial)	--	--	−3.29 (−9.75, 3.18)	−3.07 (−9.49, 3.35)
Household income as a percentage of poverty level				
≤130%	--	--	−3.63 (−9.82, 2.56)	−3.74 (−9.94, 2.46)
>130–185%	--	--	−5.88 (−13.95, 2.19)	−5.91 (−13.97, 2.16)
>185%	--	--	Referent	Referent
Constant	0.23 (0.02, 2.36)	5.73 *** (2.79, 11.81)	53.79 *** (45.68, 61.90)	55.58 *** (46.35, 64.80)

Notes: *n* = 1285 students from the 2014–2015 School Nutrition and Meal Cost Study (SNMCS). Regressions were survey-adjusted and controlled for the variables shown. Results are shown from three separate regression models: (1) a zero-inflated Poisson model examining the association of state law and school characteristics with the number of unhealthy competitive foods and beverages available, which included a logistic regression model for excess zeroes and a Poisson model for the number of unhealthy foods and beverages (shown in separate columns); (2) a linear regression model examining the association of the number of unhealthy competitive foods and beverages available and school and student characteristics with student BMI-for-age percentiles; and (3) the model in (2) with the addition of state law strength score as a covariate. CI: confidence interval. + *p* < 0.10, * *p* < 0.05, ** *p* < 0.01, *** *p* < 0.001.

## Data Availability

Requests for access to the public use SNMCS data should be submitted via electronic mail to: FNSStudies@usda.gov.
